# Pressure-Adjusted Heart Rate Predicts Mortality and Resource Utilization in Cardiogenic Shock: An Analysis of the MIMIC-IV Database

**DOI:** 10.3390/jcdd13080357

**Published:** 2026-07-29

**Authors:** Omar Khayat, Bahy Abofrekha, Mohamad B. Moumneh, Sara El Yazghi Ezzaher, Ahmed A. Zayed, Hadi Itani, Uday Sankar Akash Vankayala, Martin Miguel I. Amor

**Affiliations:** 1 Internal Medicine Department, Northwell Health, New Hyde Park, NY 10305, USA; babofrekha@northwell.edu (B.A.); mmoumneh@northwell.edu (M.B.M.); azayed@northwell.edu (A.A.Z.); hitani@northwell.edu (H.I.); uvankayala@northwell.edu (U.S.A.V.); 2 Faculté de Médecine et de Pharmacie de Fes, Fes 30000, Morocco; selyazghi2@gmail.com; 3 Cardiovascular Institute, Northwell Health, New Hyde Park, NY 10305, USA

**Keywords:** cardiogenic shock, hemodynamics, risk stratification

## Abstract

Background: Pressure-Adjusted Heart Rate (PAHR) is a novel hemodynamic index in cardiogenic shock (CS); however, its dynamic outcome remains underexplored. Methods: In this retrospective cohort study, which utilized the Medical Information Mart for Intensive Care IV (MIMIC-IV) database, PAHR was calculated using at least 12 time-matched values of mean arterial pressure (MAP), central venous pressure (CVP), and heart rate (HR) over the first 48 h. Patients receiving early mechanical circulatory support (MCS) were excluded. Multivariable regression assessed PAHR’s association with mortality, requirement of renal replacement therapy (RRT), and invasive mechanical ventilation. Effect estimates were reported per 10-unit increments of PAHR. Results: A total of 772 patients with CS were included. Mean PAHR was 15.9 ± 7.0. In our multivariable analysis, every 10-unit PAHR increase independently predicted in-hospital mortality (adjusted OR 1.97; 95% CI 1.45–2.68; *p* < 0.001). Using statistically comparable increments, PAHR showed strong association with in-hospital mortality (adjusted OR 1.97; 95% CI 1.45–2.68; *p* < 0.001) and ICU mortality (adjusted OR 1.85; 95% CI 1.35–2.54; *p* < 0.001). PAHR strongly predicted the use of RRT (aOR 2.69; *p* < 0.001) and invasive mechanical ventilation (aOR 2.11; *p* < 0.001). Among survivors, higher PAHR predicted prolonged ICU stay and vasopressor duration (*p* < 0.001). Conclusion: PAHR is a promising integrated marker of mortality and morbidity in patients with CS. It serves as a sustained predictor of outcomes, though external validation is required.

## 1. Introduction

Cardiogenic shock (CS) is a primary cardiac disorder defined by reduced cardiac output causing circulatory failure with resultant end-organ hypoperfusion and tissue hypoxia. Despite advances in pharmacologic and mechanical support strategies, it remains a major clinical challenge, with in-hospital mortality rates persistently ranging between 40% and 50% [1,2,3]. Traditionally, rapid bedside risk stratification in CS has relied on arterial-centric indices. The Shock Index (SI), defined as the ratio of heart rate (HR) to systolic blood pressure, is a validated predictor of mortality in acute myocardial infarction (AMI) and shock. However, SI has an inherent physiologic limitation as it reflects arterial hypoperfusion and compensatory tachycardia without accounting for the status of the venous circulation. As a result, it fails to capture systemic venous congestion, an important contributor to cardiorenal syndrome, hepatic dysfunction, and progressive end-organ injury [4]. In patients with the “wet and cold” phenotype of CS, where right ventricular dysfunction and volume overload are common, arterial indices alone may therefore provide an incomplete representation of the underlying hemodynamic state.

To address this limitation, pressure-adjusted heart rate (PAHR) has been proposed as a more comprehensive hemodynamic index. PAHR integrates central venous pressure (CVP) into the relationship between HR and mean arterial pressure (MAP) and is calculated as HR × (CVP/MAP) [5]. Recent studies have suggested an association between admission PAHR and mortality; however, these analyses have largely relied on single time-point measurements obtained at presentation [5]. Such static assessments are susceptible to the transient hemodynamic fluctuations associated with early resuscitation and may fail to reflect a patient’s dynamic response to therapy. Moreover, the relationship between PAHR and morbidity burden, including the need for renal replacement therapy (RRT) and invasive mechanical ventilation, remains incompletely characterized. In contemporary critical care, survival is frequently accompanied by prolonged organ support, making predictors of resource utilization and organ failure as clinically relevant as mortality itself.

In this study, we leveraged the high-resolution clinical data available in the Medical Information Mart for Intensive Care IV (MIMIC-IV) database to evaluate the prognostic utility of PAHR in patients with CS. Distinct from prior work, we employed a 48-h observation window to capture sustained hemodynamic derangement rather than transient admission physiology. We hypothesized that PAHR serves as a robust continuous predictor of both mortality and resource utilization, outperforming traditional arterial-based indices in a heterogeneous cohort of patients with CS.

## 2. Materials and Methods

### 2.1. Study Design and Population

We conducted a retrospective cohort study using the MIMIC-IV version 3.1 database. MIMIC-IV is a publicly available critical care database comprising deidentified health records from patients admitted to the Beth Israel Deaconess Medical Center between 2008 and 2022. We included adult patients aged ≥ 18 years who were admitted to the cardiac care unit (CCU) or cardiovascular intensive care unit (CVICU) for CS, identified using International Classification of Diseases (ICD-9 and ICD-10) codes. Patients were included if they had at least 12 time-matched hemodynamic measurement triplets (MAP, CVP, and HR) within the first 48 h of admission. Patients not meeting this threshold were excluded. Patients were also excluded if they received mechanical circulatory support (MCS), including intra-aortic balloon pump (IABP), Impella, or extracorporeal membrane oxygenation (ECMO).

### 2.2. Exposure: Pressure-Adjusted Heart Rate

The pressure-adjusted heart rate (PAHR) was defined mathematically as the product of heart rate and the ratio of central venous pressure to mean arterial pressure: PAHR = HR × (CVP/MAP).

To account for the asynchronous documentation of vital signs in the electronic health record, we employed a strict time-matching algorithm. For every MAP measurement recorded during the first 48 h of ICU admission, the closest CVP and HR measurements within a ±60-min window were identified to form a “time-matched triplet”. A discrete PAHR value was then calculated for each valid triplet.

To ensure a robust representation of the patient’s sustained hemodynamic state rather than a transient snapshot, inclusion in the final analysis required a minimum of 12 time-matched PAHR triplets per patient. The primary exposure variable for each patient was then defined as the arithmetic mean of all their time-matched PAHR values over the 48-h study period.

PAHR was analyzed as a continuous variable, with results expressed as odds ratios (OR) or beta coefficients (β) per 10-unit increase. This increment was chosen to align with prior literature. For descriptive purposes, patients were stratified into four groups: PAHR < 10, 10–20, 20–30, and >30.

### 2.3. Outcomes

The primary outcome was in-hospital and ICU mortality. Secondary binary outcomes included requirement for RRT (defined as the utilization of either intermittent hemodialysis or continuous renal replacement therapy (CRRT) at any point during the ICU stay), MCS, and invasive mechanical ventilation (defined as the need for endotracheal intubation) during ICU stay. Additionally, any mechanical ventilation was evaluated as a composite outcome encompassing both invasive support and non-invasive positive pressure ventilation (NIPPV, such as CPAP or BiPAP). Time-dependent continuous outcomes included ICU LOS (days) and VID (hours). These outcomes were analyzed exclusively in cohort survivors to avoid survivorship bias, as these cumulative measures are truncated by early death.

### 2.4. Covariates

Models were adjusted for 17 covariates selected a priori based on clinical relevance and prior literature. These included: demographics (age, sex); comorbidities (hypertension [HTN], coronary artery disease [CAD], heart failure with reduced ejection fraction [HFrEF], heart failure with preserved ejection fraction [HFpEF], atrial fibrillation [AF], prior myocardial infarction, diabetes mellitus [DM], chronic kidney disease [CKD] ≥ 3, and prior cardiac arrest); admission procedures (acute percutaneous coronary intervention [PCI] and coronary artery bypass grafting [CABG]); and severity markers (initial lactate, maximum lactate within 48 h, and maximum vasoactive-inotropic score [VIS] within 48 h). MAP and CVP were not included as adjustment variables to avoid collinearity, as PAHR is derived from these parameters.

### 2.5. Statistical Analysis

The statistical associations between baseline characteristics and PAHR were analyzed as a continuous variable using Spearman rank correlation for continuous variables and the Mann–Whitney U test for binary categorical variables. Baseline characteristics analyzed were reported in categorical bins for visual representation. Univariate regression analyses were first performed to assess the crude association between PAHR and each outcome. Multivariable logistic regression was then used for binary outcomes, and multivariable linear regression for continuous outcomes, adjusting for all 17 covariates. Effect estimates were reported as OR with 95% confidence intervals (CIs) for binary outcomes and β coefficients with 95% CI for continuous outcomes, per 10-unit increase in PAHR. Model discrimination for mortality was assessed using the area under the receiver operating characteristic curve (AUC). A two-sided *p*-value < 0.05 was considered statistically significant. All analyses were performed using Python (version 3.13) with the statsmodels and scipy libraries.

### 2.6. Temporal Analysis of PAHR Trajectories

To address whether averaging PAHR across 48 h obscures prognostically important hemodynamic shifts, we divided the observation period into an admission window (0–12 h) and a late window (36–48 h), averaging PAHR within each. Patients required at least three time-matched triplets per window; those receiving mechanical circulatory support within 48 h were excluded, as in the primary analysis. Paired values were compared using the Wilcoxon signed-rank test, and the correlation between windows assessed by Pearson correlation.

Patients presenting with a high PAHR tend to fall over the first 48 h and those presenting low tend to rise, so the later value cannot be interpreted without reference to the admission value. Multivariable logistic regression (binary outcomes) and linear regression (continuous outcomes) therefore included both windows together with the same 16 covariates as the primary analysis, so that the later value was assessed relative to what would be expected given each patient’s own admission PAHR. Estimates are reported as adjusted odds ratios or β coefficients per 5-unit increase. The same association was additionally examined within admission PAHR categories (<10, 10–20, 20–30, >30), with a likelihood ratio test for interaction between the late value and admission category.

To determine whether measuring PAHR in separate windows improved prediction over the aggregate exposure used in the primary analysis, we compared the discrimination (area under the receiver operating characteristic curve) of three logistic models for mortality: the aggregate 48-h mean, admission PAHR alone, and admission plus late PAHR. Differences in area under the curve against the aggregate mean were estimated by paired bootstrap resampling (2000 replicates), and the nested comparison of the two-window model against admission alone was assessed using a likelihood ratio test.

## 3. Results

A total of 772 patients with CS admitted to the CCU or CVICU were included in the final analysis. Mean age was 66.9 ± 12.7 years. Of the total population, 68.0% (n = 525) were male. The cohort carried a high burden of comorbidities, including HTN (75.9%), CAD (62.6%), HFrEF (53.2%), and AF (42.7%). AMI was present in 218 (28.2%) patients. Among the AMI-CS cohort, acute revascularization was performed in 159 (72.9%) patients, with 28.9% undergoing PCI and 48.2% undergoing CABG. Baseline characteristics are detailed in Table 1.

The data reveal a distinct, progressive physiological deterioration as the composite PAHR index worsens. Specifically, as patients transition from the lowest (<10) to the highest (>30) PAHR strata, the isolated hemodynamic components diverge significantly: mean arterial pressure (MAP) steadily declines (74.9 to 67.5 mmHg), while both central venous pressure (CVP) and heart rate (HR) markedly increase (7.4 to 23.6 mmHg and 79.5 to 107.1 bpm, respectively; all continuous correlation *p* < 0.001). This simultaneous burden of worsening hypotension, progressive venous congestion, and severe compensatory tachycardia is also mirrored metabolically, with serum lactate levels rising significantly in tandem with higher PAHR scores (*p* < 0.001). Together, these findings demonstrate how an elevated PAHR accurately consolidates the severity of both hemodynamic and metabolic derangement in cardiogenic shock.

**Figure 1:**
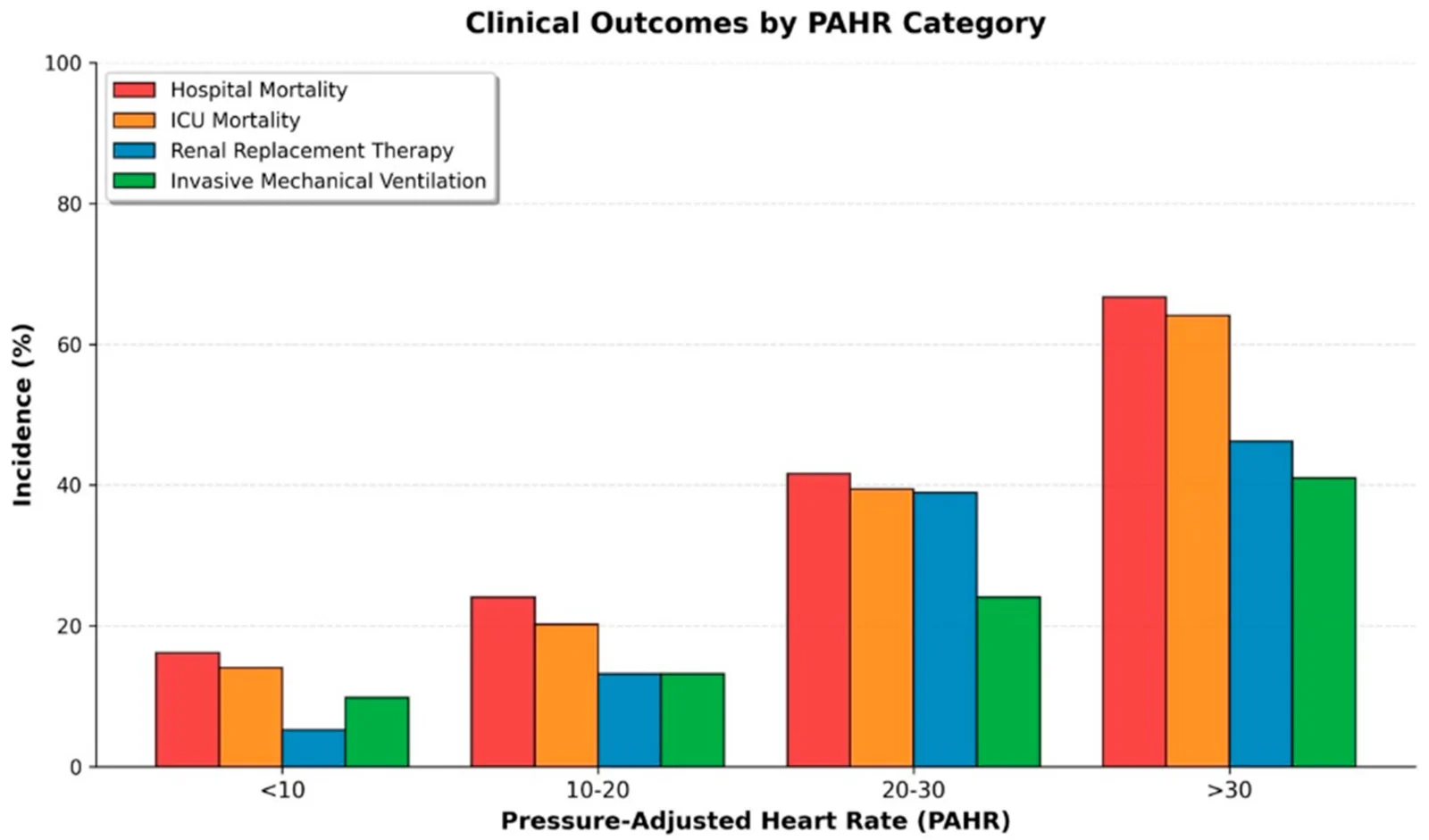
Clinical Outcomes by PAHR Category.

The mean PAHR across the entire cohort was 15.9 ± 7.0. Patients were grouped into four descriptive categories: PAHR < 10 (17.6%), PAHR 10–20 (60.2%), PAHR 20–30 (18.7%), and PAHR > 30 (3.5%). Elevated PAHR was independently associated with major organ failure and renal dysfunction. In multivariable analysis, PAHR predicted both RRT requirement (adjusted OR 2.69; 95% CI 1.97–3.67; *p* < 0.001) and need for invasive mechanical ventilation (adjusted OR 2.11; 95% CI 1.57–2.84; *p* < 0.001). Furthermore, PAHR showed an inverse linear relationship with urine output (adjusted β −0.14 mL/kg/h per 10-unit increase; *p* < 0.001). No significant association was observed for MCS requirements (Figure 1).

Overall, in-hospital mortality was 25.3% and ICU mortality was 22.0%. Mortality rates demonstrated a significant stepwise rise with higher PAHR (Table 2). In univariate analysis, every 10-unit increase in PAHR was associated with higher odds of in-hospital mortality (OR 2.20; 95% CI 1.73–2.79; *p* < 0.001) and ICU mortality (OR 1.85; 95% CI 1.35–2.54; *p* < 0.001). After adjustment, PAHR remained a robust independent predictor of in-hospital mortality (adjusted OR 1.97; 95% CI 1.45–2.68; *p* < 0.001) and ICU mortality (adjusted OR 1.85; 95% CI 1.35–2.54; *p* < 0.001) (Table 3). The prognostic value of PAHR varied by shock etiology. In the non-AMI-CS cohort (n = 554), PAHR remained a robust independent predictor of in-hospital mortality (adjusted OR 2.36; 95% CI 1.61–3.45; *p* < 0.001). In the AMI-CS subgroup (n = 218), while mortality rates trended higher with increasing PAHR, the association did not reach statistical significance after multivariable adjustment (adjusted OR 1.14; 95% CI 0.58–2.25; *p* = 0.70). In multivariable analysis of the AMI-CS cohort, the only factors significantly associated with in-hospital mortality were advanced age (aOR 1.05; 95% CI 1.03–1.08; *p* < 0.001), peak lactate levels (aOR 1.35; 95% CI 1.12–1.63; *p* = 0.002), and revascularization strategy, with both acute PCI (aOR 0.38; 95% CI 0.16–0.89; *p* = 0.025) and acute CABG (aOR 0.08; 95% CI 0.03–0.21; *p* < 0.001) conferring a survival benefit. Among ICU survivors (n = 602), higher PAHR was associated with significant resource utilization, including prolonged ICU length of stay (adjusted β +3.46 days per 10-unit increase; *p* < 0.001) and longer duration of vasoactive-inotropic support (adjusted β +58.3 h per 10-unit increase; *p* < 0.001) (Table 3).

**Figure 2:**
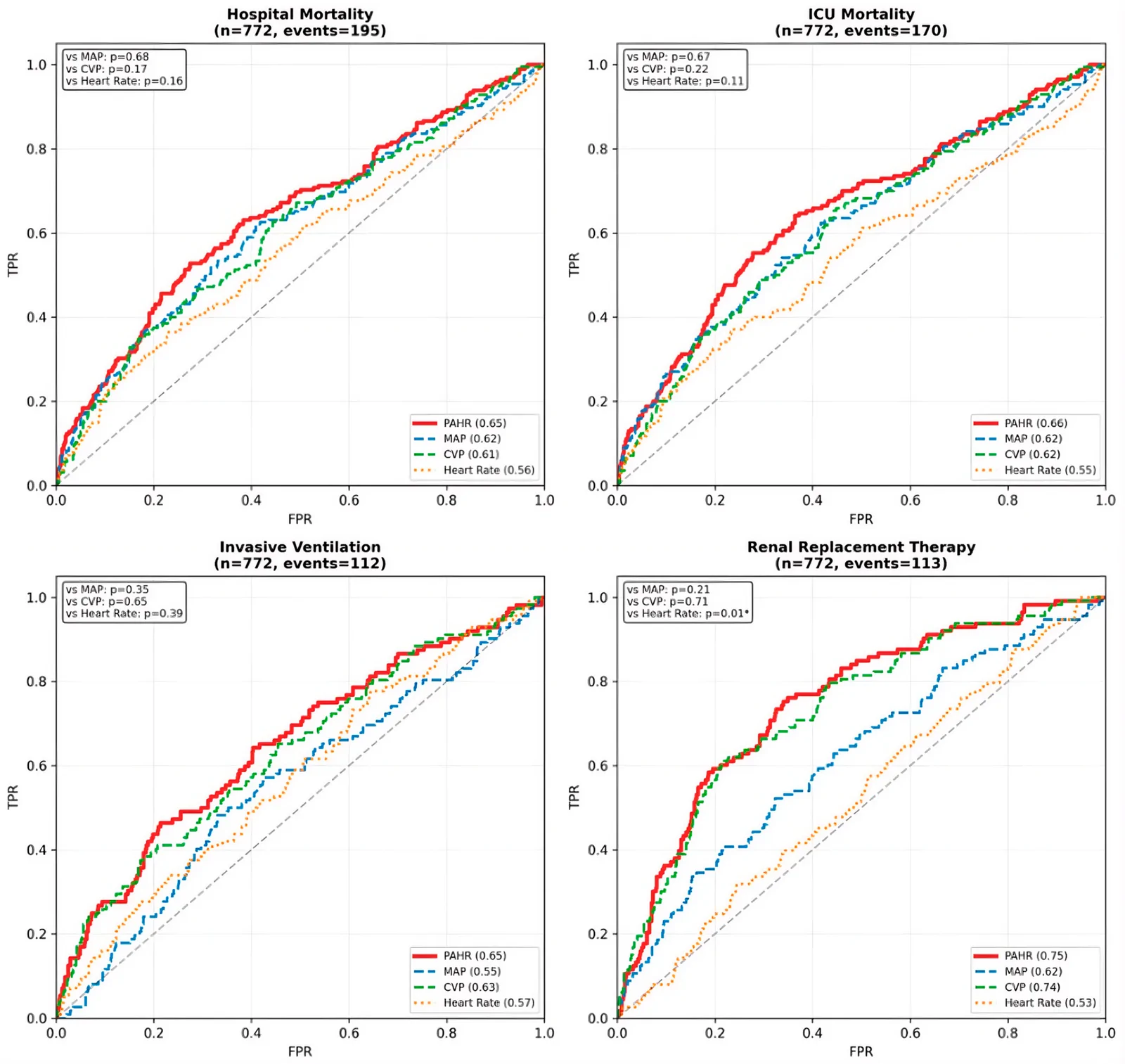
Receiver Operating Characteristic (ROC) Curves for Clinical Outcomes. Discriminative performance of Pressure-Adjusted Heart Rate (PAHR) compared to its individual hemodynamic components for the prediction of hospital mortality, ICU mortality, invasive mechanical ventilation, and renal replacement therapy. Pairwise statistical comparisons of AUCs were performed using DeLong’s test, with PAHR serving as the reference standard. * *p* < 0.05.

There was a distinct stepwise deterioration in hemodynamics across increasing PAHR categories (Table 1). As PAHR increased, MAP decreased, while CVP and HR significantly increased (*p* < 0.001). These hemodynamic derangements correlated with markers of metabolic shock severity. Initial lactate, maximum 48-h lactate, and maximum vasoactive-inotropic score (VIS) all increased significantly with higher PAHR (*p* < 0.001). We evaluated the prognostic performance of PAHR relative to its individual components using clinical increments that represented comparable statistical deviations within our cohort (~1.3–1.4 SD). Specifically, we compared a 10-unit increase in PAHR against a 10 mmHg decrease in MAP, a 6 mmHg increase in CVP, and a 20 bpm increase in HR. For the prediction of in-hospital mortality, PAHR demonstrated a stronger association (aOR 1.97) compared to MAP (aOR 1.47; *p* = 0.013), CVP (aOR 1.36; *p* = 0.028), and HR (aOR 1.62; *p* < 0.001). For RRT prediction, while CVP (aOR 2.72; *p* < 0.001) was the strongest individual component predictor, the integrated PAHR index provided comparable risk estimation (aOR 2.69; *p* < 0.001). PAHR outperformed individual components for invasive mechanical ventilation (aOR 2.11 vs. CVP 1.85, HR 1.46, MAP non-significant) and urine output preservation (Table 4). We also assessed the discriminative ability of PAHR compared to its components using receiver operating characteristic (ROC) curve analysis. For the prediction of in-hospital mortality, PAHR achieved an area under the curve (AUC) of 0.646, which was statistically comparable to MAP (AUC 0.618), CVP (AUC 0.610), and heart rate (AUC 0.564) (*p* > 0.05). Notably, PAHR demonstrated robust discrimination for the prediction of RRT (AUC 0.751), performing comparably to CVP (AUC 0.736; *p* = 0.70) and MAP (AUC 0.622; *p* = 0.21), while significantly outperforming HR (AUC 0.532; *p* = 0.012) (Figure 2).

### Temporal Trajectory and Discriminative Performance

A total of 475 patients had sufficient time-matched PAHR measurements in both windows and were included in the temporal cohort. Patients dying before 36 h could not contribute. PAHR declined modestly between windows (admission 16.6 ± 6.9 vs. 36–48 h 15.6 ± 7.1; median change −1.5, IQR [−4.7; +2.7]; *p* < 0.001). The two values were correlated within patients (r = 0.55) and the magnitude of change did not differ between hospital survivors and non-survivors (median −1.6 versus −0.9, *p* = 0.73).

After adjusting for the baseline admission value, a higher PAHR at 36–48 h was not associated with hospital mortality (aOR 1.15 per 5-unit increase, 95% CI 0.94–1.41) or ICU mortality (aOR 1.15 per 5-unit increase; 95% CI 1.11, 0.90–1.37), but was independently associated with renal replacement therapy (1.47, 1.19–1.82), invasive mechanical ventilation (1.24, 1.02–1.52), and lower urine output (β −0.09 mL/kg/h, −0.14 to −0.04). These associations were consistent across admission PAHR categories, with no evidence of effect modification (interaction *p* = 0.45, 0.49 and 0.87, respectively).

Separating PAHR into discrete windows did not improve mortality prediction relative to the aggregate 48-h mean (Table 5). Neither admission PAHR alone nor the two-window model outperformed the aggregate mean for hospital or ICU mortality, and adding the late window to admission PAHR conferred no benefit.

## 4. Discussion

PAHR was first introduced by Ginder et al. as a novel composite index that captures three distinct variables of hemodynamic failure simultaneously: compensatory chronotropic stress, arterial hypoperfusion, and systemic venous congestion., with each parameter providing valuable information. For HR, Jentzer et al. demonstrated that compensatory tachycardia mirrors shock severity, with HRs rising from 85 bpm in SCAI Stage C (16.2% mortality) to 95 bpm in Stage E (67.0% mortality) (*p*-values < 0.001) [1]. As stroke volume deteriorates, cardiac output becomes progressively rate-dependent, positioning HR as a vital marker of physiologic stress. For MAP, Parlow et al. identified a MAP < 70 mmHg during the first 36 h as a threshold for adverse outcomes, noting that patients maintained below this level exhibited significantly higher mortality [6]. Similarly, Burstein et al. reported a graded inverse relationship between mean MAP and in-hospital mortality (adjusted OR 0.9 per 5 mmHg increase), with risks persisting even in patients meeting the standard ≥ 65 mmHg target [7]. As for CVP, Chen et al.’s meta-analysis demonstrated that elevated CVP is associated with two-fold increased odds of acute kidney injury (OR 2.22), driven by increased renal afterload and impaired trans-renal pressure gradients [8]. By quantifying tachycardia, hypotension, and congestion, PAHR identifies the highest-risk phenotype and preserves the simplicity of a bedside ratio, thereby distinguishing the cold and wet phenotype that arterial markers alone often miss. This represents a critical evolution from the traditional Shock Index (HR/SBP) that is restricted to arterial hemodynamics and remains blind to the venous circulation [4].

Despite this physiological rationale, it is important to acknowledge that in our discriminative analyses, the integrated PAHR index did not demonstrate statistical superiority (via AUC) over its individual hemodynamic components, such as MAP or CVP alone. However, the clinical value of PAHR does not necessarily depend on outperforming isolated metrics mathematically, but rather on its capacity for holistic integration. Relying on a single hemodynamic parameter can be clinically misleading; for instance, a patient may exhibit a deceptively “adequate” MAP that is entirely dependent on extreme compensatory tachycardia, or they may have severe, unrecognized venous congestion driving renal failure despite a normal arterial profile. By consolidating chronotropic stress, arterial perfusion, and venous congestion into a single bedside metric, PAHR prevents the clinician from anchoring on a single reassuring value, providing a more balanced representation of the true shock phenotype.

Ginder et al.’s work demonstrated a stepwise mortality gradient across PAHR categories and reported a 35% increase in adjusted mortality odds per 10-unit increment [5]. While their work established PAHR as a promising prognostic marker for in-hospital mortality using admission values, the significance of its temporal dynamics and its predictive value for renal preservation and resource utilization remain unexplored. In our cohort, elevated PAHR was significantly associated with higher in-hospital and ICU mortality, the need for RRT, invasive mechanical ventilation, and reduced urine output in both unadjusted and adjusted analyses. PAHR was also associated with prolonged ICU length of stay and extended VID, which remained significant in survivor-restricted sensitivity analyses. Our findings corroborate and extend the observations of Ginder et al., revealing important temporal distinctions in PAHR’s prognostic significance. While mortality rates in our cohort were comparable to Ginder et al.’s across lower PAHR categories (15.4% vs. 14.0% for PAHR < 10), they markedly diverged in the highest-risk group. Patients in our cohort with PAHR ≥ 30 demonstrated a mortality of 66.7%, nearly double the 37.6% reported by Ginder et al. This striking difference highlights the superior discriminative capacity of persistent PAHR elevation. Unlike a single admission snapshot, which often captures transient instability, sustained elevation isolates the cohort with resistant shock. Consequently, PAHR functions as a robust identifier of non-responders. Beyond mortality, PAHR was strongly associated with morbidity, most notably regarding the need for RRT. This finding is mechanistically linked to resource use: as the FRENSHOCK study demonstrated, patients requiring RRT represent a high-acuity phenotype that almost invariably requires more invasive ventilation and aggressive hemodynamic support [9]. By identifying the hemodynamic precursors to renal failure, PAHR effectively flagged this resource-intensive phenotype, serving as a significant independent predictor for invasive mechanical ventilation, extended vasopressor duration, and prolonged ICU length of stay. Prognostic utility diverged significantly by etiology. While PAHR remained a robust independent predictor of mortality in the non-AMI cohort (aOR 2.36, *p* < 0.001), it was not predictive in the AMI-CS subgroup. This suggests that in ischemic shock, the profound impact of reperfusion overrides the prognostic signal of initial hemodynamics. Other factors significantly associated with mortality included advanced age and peak lactate levels. These findings align with Davierwala et al., who identified age and lactate as the strongest predictors of mortality in patients undergoing emergency revascularization for infarct-related shock [10].

## 5. Limitations

This study has several limitations.

1. As a single-center retrospective analysis using the MIMIC-IV database, external validation is warranted.
2. We excluded patients receiving mechanical circulatory support within the first 48 h to avoid confounding from device-induced hemodynamic alterations, which limits generalizability to the population receiving immediate MCS.
3. Requiring at least 12 time-matched hemodynamic triplets within 48 h creates a highly selected cohort, introducing monitoring and survivorship bias by overrepresenting patients who required continuous invasive monitoring and survived the initial 48 h.
4. While our time-matching algorithm minimized error, residual variability in measurement intervals may influence PAHR calculations.
5. Our multivariable models adjusted for maximum lactate and maximum vasoactive-inotropic score (VIS) measured within the same 48-h window as our PAHR calculations. This concurrent timing introduces a potential risk of overadjustment, as these variables may represent downstream clinical deterioration rather than independent baseline confounders, which could result in overly conservative effect estimates for PAHR.
6. Patients with baseline end-stage renal disease (ESRD) were not explicitly excluded. While baseline renal dysfunction was adjusted for statistically using the chronic kidney disease/ESRD covariate, RRT utilization in the ESRD subgroup likely reflects maintenance therapy rather than incident acute kidney injury.
7. Our analysis utilized a single aggregate mean PAHR across the first 48 h to represent the overall hemodynamic burden. Our study design did not incorporate specific time windows or serial assessments to evaluate temporal trends. Consequently, we are unable to assess PAHR variability or calculate a delta-PAHR, which limits our ability to differentiate between distinct physiological trajectories. Future studies incorporating discrete time-window analyses are needed to determine whether evaluating the dynamic trajectory of PAHR offers incremental prognostic value over a single aggregate mean.
8. Due to the retrospective nature of the MIMIC-IV database, we were unable to reliably differentiate the exact etiology of cardiogenic shock (e.g., acute myocardial infarction-related shock versus acute-on-chronic non-ischemic heart failure). Administrative billing codes lack the granular clinical data necessary to definitively adjudicate shock etiology without the risk of significant misclassification.
9. Our cohort exhibited an unusually high CABG-to-PCI ratio. Since we did not explicitly exclude patients undergoing cardiac surgery, we strongly suspect our dataset includes a high representation of post-cardiotomy cardiogenic shock. This confounding presence likely explains the high CABG rate and limits the generalizability of our findings to strictly medical cardiac care populations.
10. The optimal PAHR thresholds for clinical decision-making require prospective validation, as our categorical stratifications were derived for descriptive purposes.

## 6. Conclusions

Pressure-adjusted heart rate (PAHR) is a practical bedside metric that captures circulatory failure in cardiogenic shock by consolidating arterial, venous, and chronotropic data. Although it did not yield significantly higher discrimination than its individual parameters, PAHR serves as a robust longitudinal marker for predicting mortality, morbidity, and intense resource utilization. Prospective multicenter studies are necessary to confirm its clinical utility and safely guide patient management.

## Figures and Tables

**Table 1:** Baseline Characteristics Stratified by PAHR.

Variable	Overall (N = 772)	PAHR < 10 (N = 136)	PAHR 10–20 (N = 465)	PAHR 20–30 (N = 144)	PAHR > 30 (N = 27)	*p*-Value (with Continuous PAHR)
DEMOGRAPHICS						
Age, years (Mean ± SD)	66.9 ± 12.7	68.6 ± 11.6	66.8 ± 12.9	65.8 ± 13.1	66.4 ± 13.1	0.401
Male Sex, No. (%)	525 (68.0%)	101 (74.3%)	315 (67.7%)	87 (60.4%)	22 (81.5%)	0.016
COMORBIDITIES, No. (%)						
Acute Myocardial Infarction	218 (28.2%)	46 (33.8%)	131 (28.2%)	39 (27.1%)	2 (7.4%)	0.007
Hypertension	586 (75.9%)	110 (80.9%)	350 (75.3%)	106 (73.6%)	20 (74.1%)	0.136
Coronary Artery Disease	483 (62.6%)	93 (68.4%)	295 (63.4%)	82 (56.9%)	13 (48.1%)	0.010
Heart Failure (HFrEF)	411 (53.2%)	72 (52.9%)	261 (56.1%)	67 (46.5%)	11 (40.7%)	0.091
Heart Failure (HFpEF)	117 (15.2%)	24 (17.6%)	63 (13.5%)	24 (16.7%)	6 (22.2%)	0.475
Atrial Fibrillation	330 (42.7%)	53 (39.0%)	189 (40.6%)	69 (47.9%)	19 (70.4%)	0.051
Prior Myocardial Infarction	257 (33.3%)	52 (38.2%)	157 (33.8%)	45 (31.2%)	3 (11.1%)	0.020
Diabetes Mellitus	313 (40.5%)	46 (33.8%)	188 (40.4%)	66 (45.8%)	13 (48.1%)	0.025
Chronic Kidney Disease	284 (36.8%)	44 (32.4%)	166 (35.7%)	62 (43.1%)	12 (44.4%)	0.018
Prior Cardiac Arrest	87 (11.3%)	12 (8.8%)	55 (11.8%)	16 (11.1%)	4 (14.8%)	0.093
ADMISSION PROCEDURES, No. (%)						
PCI during admission	80 (10.4%)	21 (15.4%)	45 (9.7%)	14 (9.7%)	0 (0.0%)	0.038
CABG during admission	210 (27.2%)	39 (28.7%)	141 (30.3%)	28 (19.4%)	2 (7.4%)	0.015
HEMODYNAMICS (First 48 h)						
PAHR Index (HR × CVP/MAP)	15.9 ± 7.0	7.7 ± 1.7	14.4 ± 2.6	24.0 ± 2.8	37.4 ± 6.6	<0.001
MAP, mmHg	72.5 ± 7.0	74.9 ± 6.6	72.6 ± 6.5	70.7 ± 7.9	67.5 ± 6.9	<0.001
CVP, mmHg	12.6 ± 4.5	7.4 ± 2.0	12.0 ± 2.5	17.5 ± 3.1	23.6 ± 4.2	<0.001
Heart Rate, bpm	88.8 ± 15.3	79.5 ± 12.6	87.9 ± 13.4	97.3 ± 15.8	107.1 ± 18.1	<0.001
SEVERITY & METABOLIC						
Initial Lactate, mmol/L	3.2 ± 2.6	2.4 ± 1.6	3.1 ± 2.3	4.3 ± 3.7	3.7 ± 2.4	<0.001
Max Lactate (48 h), mmol/L	5.1 ± 3.8	3.4 ± 2.4	5.0 ± 3.5	6.5 ± 4.8	8.3 ± 5.0	<0.001
Max VIS Score (48 h)	49.0 ± 143.7	17.8 ± 57.1	45.4 ± 157.9	81.7 ± 149.7	94.8 ± 115.4	<0.001

Abbreviations: CABG, coronary artery bypass grafting; CVP, central venous pressure; HFpEF, heart failure with preserved ejection fraction; HFrEF, heart failure with reduced ejection fraction; MAP, mean arterial pressure; PAHR, pressure-adjusted heart rate; PCI, percutaneous coronary intervention; SD, standard deviation; VIS, vasoactive-inotropic score. Note: While patients are grouped into categorical PAHR bins for visual representation, *p*-values denote the statistical association between the respective baseline variable and PAHR evaluated as a continuous variable across the entire cohort. Associations were tested using Spearman rank correlation for continuous variables and the Mann–Whitney U test for binary categorical variables.

**Table 2:** Clinical Outcomes and Resource Utilization Stratified by PAHR Category.

Outcome	Overall (N = 772)	PAHR < 10 (N = 136)	PAHR 10–20 (N = 465)	PAHR 20–30 (N = 144)	PAHR > 30 (N = 27)
Hospital Mortality	195 (25.3%)	21 (15.4%)	104 (22.4%)	52 (36.1%)	18 (66.7%)
ICU Mortality	170 (22.0%)	18 (13.2%)	88 (18.9%)	47 (32.6%)	17 (63.0%)
Renal Replacement Therapy	113 (14.6%)	7 (5.1%)	44 (9.5%)	50 (34.7%)	12 (44.4%)
Invasive Mechanical Ventilation	112 (14.5%)	11 (8.1%)	54 (11.6%)	36 (25.0%)	11 (40.7%)
Any Mechanical Ventilation	666 (86.3%)	115 (84.6%)	402 (86.5%)	125 (86.8%)	24 (88.9%)
MECHANICAL CIRCULATORY SUPPORT					
Any MCS *	35 (4.5%)	5 (3.7%)	26 (5.6%)	3 (2.1%)	1 (3.7%)
IABP	27 (3.5%)	4 (2.9%)	20 (4.3%)	2 (1.4%)	1 (3.7%)
Impella	7 (0.9%)	1 (0.7%)	5 (1.1%)	1 (0.7%)	0 (0.0%)
ECMO	2 (0.3%)	1 (0.7%)	1 (0.2%)	0 (0.0%)	0 (0.0%)
RESOURCE UTILIZATION					
ICU Length of Stay, days	8.5 ± 9.4	5.8 ± 5.7	8.5 ± 9.1	11.2 ± 12.2	7.9 ± 8.1
Vasopressors/Intoropes Duration, hours	75.9 ± 141.3	29.8 ± 56.6	69.1 ± 132.3	135.5 ± 197.4	105.4 ± 126.8
Urine Output, mL/kg/h	1.0 ± 0.6	1.1 ± 0.6	1.1 ± 0.6	0.8 ± 0.7	0.6 ± 0.6

Abbreviations: PAHR, pressure-adjusted heart rate; IABP, intra-aortic balloon pump; ECMO, Extracorporeal Membrane Oxygenation. * Note: Patients receiving MCS within the first 48 h of admission were excluded from the study cohort. MCS rates reported here represent rescue or late device utilization occurring after the initial 48-h window.

**Table 3:** Association Between 10-Unit Increase in PAHR and Outcomes.

Binary Outcome	Overall Cohort (N = 772)	Unadjusted Estimate Odds Ratio	Adjusted Estimate * Odds Ratio [95% CI]	*p*-Value
Hospital Mortality	195 (25.3%)	2.20	1.97 [1.45–2.68]	<0.001
ICU Mortality	170 (22.0%)	2.26	1.85 [1.35–2.54]	<0.001
Renal Replacement Therapy	113 (14.6%)	2.96	2.69 [1.97–3.67]	<0.001
Invasive Mechanical Ventilation	112 (14.5%)	2.07	2.11 [1.57–2.84]	<0.001
Mechanical Ventilation (invasive + non-invasive)	666 (86.3%)	1.11	0.78 [0.54–1.14]	0.201
Any Rescue MCS Required	35 (4.5%)	0.95	1.22 [0.69–2.17]	0.497
IABP Required	27 (3.5%)	0.88	1.33 [0.68–2.59]	0.403
Impella Required	7 (0.9%)	−	‡	−
ECMO Required	2 (0.3%)	−	‡	−
Continuous Outcomes	Mean ± SD	Unadjusted Diff ß	Adjusted Diff ß *	*p*-Value
ICU Length of Stay (days) †	8.5 ± 9.4	4.2	3.5 [2.3 to 4.6]	<0.001
Vasopressors/ Inotropes Duration (hours) †	75.9 ± 141.3	65.8	58.3 [42.8 to 73.8]	<0.001
Urine Output (mL/kg/h)	1.0 ± 0.6	−0.19	−0.14 [−0.21 to −0.07]	<0.001

Abbreviations: MCS, Mechanical Circulatory Support; IABP, Intra-aortic Balloon Pump; RRT, Renal Replacement Therapy; VIS, Vasoactive-Inotropic Score. * Adjusted for age, sex, comorbidities (HTN, CAD, HFrEF, HFpEF, AFib, MI, DM, CKD, Cardiac Arrest), admission procedures (PCI, CABG), lactate, and VIS score. † Restricted to ICU survivors (N = 602) to avoid survivor bias. ‡ Regression analysis not performed due to low event counts (<10 events).

**Table 4:** Adjusted Odds Ratios of PAHR vs. MAP, CVP, and Heart Rate.

Outcome	PAHR (per 10 Units ↑)	MAP (per 10 mmHg ↓)	CVP (per 5 mmHg ↑)	Heart Rate (per 20 bpm ↑)
Hospital Mortality	1.97 (1.45–2.68) *p* < 0.001	1.47 (1.09–2.00) *p* = 0.013	1.36 (1.03–1.78) *p* = 0.028	1.62 (1.25–2.11) *p* < 0.001
ICU Mortality	1.85 (1.35–2.54) *p* < 0.001	1.52 (1.09–2.08) *p* = 0.014	1.32 (1.00–1.75) *p* = 0.053	1.48 (1.13–1.95) *p* = 0.005
RRT Requirement	2.69 (1.97–3.67) *p* < 0.001	1.56 (1.12–2.17) *p* = 0.008	2.72 (2.02–3.68) *p* < 0.001	1.17 (0.88–1.57) *p* = 0.280
Invasive Mechanical Ventilation	2.11 (1.57–2.84) *p* < 0.001	1.11 (0.81–1.54) *p* = 0.511	1.85 (1.40–2.44) *p* < 0.001	1.46 (1.11–1.94) *p* = 0.008
Urine Output (mL/kg/h)	−0.14 (−0.21 to −0.07) *p* < 0.001	−0.09 (−0.16 to −0.01) *p* = 0.019	−0.10 (−0.17 to −0.04) *p* = 0.002	−0.03 (−0.09 to 0.03) *p* = 0.333

Abbreviations: PAHR, Pressure-Adjusted Heart Rate; MAP, Mean Arterial Pressure; CVP, Central Venous Pressure; RRT, Renal Replacement Therapy.

**Table 5:** Discrimination of windowed PAHR models versus the aggregate 48-h mean (n = 475).

Model	Hospital Mortality			ICU Mortality		
	AUC	Δ (95% CI)	*p*	AUC	Δ (95% CI)	*p*
Aggregate 48-h mean	0.801	reference	—	0.792	reference	—
Admission alone (0–12 h)	0.798	−0.003 (−0.010 to +0.003)	0.30	0.790	−0.002 (−0.009 to +0.003)	0.44
Admission + late (36–48 h)	0.801	−0.000 (−0.005 to +0.005)	0.99	0.792	+0.000 (−0.004 to +0.004)	0.96

All models adjusted for the same 16 covariates as the primary analysis. ΔAUC represents the difference in area under the curve compared to the aggregate 48-h mean reference model, calculated from 2000 paired bootstrap resamples.

## Data Availability

The original contributions presented in this study are included in the article. Further inquiries can be directed to the corresponding author.
